# Optimal Nitrate Supplementation in *Phaeodactylum tricornutum* Culture Medium Increases Biomass and Fucoxanthin Production

**DOI:** 10.3390/foods11040568

**Published:** 2022-02-16

**Authors:** Clélia Afonso, Ana Rita Bragança, Bárbara A. Rebelo, Tânia S. Serra, Rita Abranches

**Affiliations:** 1 MARE—Marine and Environmental Sciences Centre, ESTM, Polytechnic of Leiria, Edifício CETEMARES, Avenida Porto de Pesca, 2520-641 Peniche, Portugal; clelia@ipleiria.pt; 2 ITQB NOVA—Instituto de Tecnologia Química e Biológica António Xavier, Universidade Nova de Lisboa, Avenida República, 2780-157 Oeiras, Portugal; rita.braganca@itqb.unl.pt (A.R.B.); brebelo@itqb.unl.pt (B.A.R.); tania.serra@itqb.unl.pt (T.S.S.)

**Keywords:** microalgae, model diatom, carotenoid, fucoxanthin optimisation, nitrate supplementation

## Abstract

*Phaeodactylum tricornutum* is a model diatom with numerous potential applications in the industry, including the production of high-value carotenoid pigments such as fucoxanthin. This compound is a potent antioxidant currently extracted mainly from brown macroalgae. Fucoxanthin exhibits several biological properties with well-known beneficial effects in the treatment and prevention of lifestyle-related diseases. *P. tricornutum* offers a valuable alternative to macroalgae for fucoxanthin production as it has a specific productivity that is 10-fold higher as compared with macroalgae. However, production processes still need to be optimised to become a cost-effective alternative. In this work, we investigated the optimal supplementation of nitrate in a cultivation medium that is currently used for *P. tricornutum* and how this nitrate concentration affects cell growth and fucoxanthin production. It has previously been shown that the addition of sodium nitrate increases productivity, but optimal conditions were not accurately determined. In this report, we observed that the continuous increase in nitrate concentration did not lead to an increase in biomass and fucoxanthin content, but there was rather a window of optimal values of nitrate that led to maximum growth and pigment production. These results are discussed considering both the scale up for industrial production and the profitability of the process, as well as the implications in the cell’s metabolism and effects in fucoxanthin production.

## 1. Introduction

Carotenoids are high-value pigments produced by photosynthetic organisms with several benefits in terms of health and nutrition. These molecules are important antioxidants, and their applications range from animal feed to human food and dietary supplements, cosmetics and pharmaceuticals [1,2,3]. The global carotenoid market is expected to have a steady increase of 5% during the next decade, with carotenoid sales likely to be over 2 billion US Dollars by 2031 [4]. Because humans and animals are unable to synthesise carotenoids and thus must ingest them through diet [5], carotenoids are often included in food and feed supplements to improve human and animal health and increase the product nutritional value. To date, carotenoid natural producers cannot keep up with market demands since the extraction and yield of these pigments still needs improvement. For this reason, carotenoids for animal feeding are obtained by chemical synthesis, often using precursors derived from the petrochemical industry [6,7]. In the case of compounds intended for human consumption, there is a growing demand for carotenoids obtained from nature; thus, innovative methods favouring natural sources should be sought, towards more sustainable production pipelines.

In order to face this challenge, two approaches are possible, either using natural producers—such as microalgae, as the ones used in this study—which already contain the necessary pathways but lack optimisation for higher yields, or non-natural heterologous systems which can be modified through metabolic engineering to produce the desired carotenoids [8,9]. Metabolic engineering of non-carotenogenic bacteria and yeast has achieved higher production yields and reduced production costs [10]. The introduction of carotenoid biosynthetic genes in specific combinations and arrangements was shown to greatly increase the production of carotenoids in *Escherichia coli* [9,11]. Zeaxanthin production was achieved by introducing a gene cluster from the bacterium *Pantoea ananatis* [12], whereas violaxanthin was achieved through the combination of the *Pantoea ananatis* gene cluster with specific genes from *Haematococcus pluvialis* or *Capsicum annuum* [13]. *Saccharomyces cerevisiae* has also been engineered by the introduction of gene clusters from various sources for the heterologous production of carotenoids, such as lycopene [14], β-carotene [15] and violaxanthin [16]. On the other hand, genetic engineering of natural producers such as microalgae has also shown to be an effective approach to increase carotenoid production. The overexpression of endogenous genes involved in the carotenoid biosynthesis pathway, such as the *phytoene synthase* (*PSY*) or the *1-deoxy-D-xylulose 5-phosphate synthase* (*DXS*) gene, increased fucoxanthin content in *Phaeodactylum tricornutum* [17,18]. In this species, the overexpression of *violaxanthin de-epoxidase* (*Vde*), *vde-related* (*Vdr*) and/or *zeaxanthin epoxidase 3* (*Zep3*) gene combinations also originated modifications in the carotenoid pattern of accumulation [19]. *Chlamydomonas reinhardtii* knockout mutants for a *ZEP* gene greatly increased the production of zeaxanthin without reducing the lutein content [20].

Despite these efforts, industry stakeholders resist accepting and implementing genetic modification solutions, mainly due to constraints associated with organism containment. Thus, microalgae, which are recognised as natural producers of several bioactive compounds with high yield, may prove to be the obvious alternative. In view of this, we devised a strategy for the optimisation of the culture conditions of the brown microalgae *P. tricornutum* for the enhanced production of fucoxanthin. This pigment offers remarkable biological properties, which are based on its unique molecular structure that is responsible for its high antioxidant activity. Indeed, many of its biological effects are related to the ability to scavenge reactive oxygen species [21,22]. Fucoxanthin has a high commercial value as a potential health promoter, not only due to its antioxidant activity, but also because of anticancer, anti-inflammatory and anti-obesity effects and other valuable properties [22]. These properties point to fucoxanthin as a potential food ingredient, important for the functional and nutraceutical properties it can bring to foods [23].

*P. tricornutum* is a promising source of fucoxanthin, since it contains at least ten times more fucoxanthin per gram of dry weight than brown algae [2], but the optimisation of yields needs to be further achieved. The capacity of these microalgae to grow under mixotrophic cultivation, i.e., combining photoautotrophic and heterotrophic growth, increases the flexibility in cultivation conditions and leads to higher biomass concentrations compared with photoautotrophic conditions [24]. The impact of light intensity and quality, as well as medium composition, are examples of parameters that have been studied and shown to affect fucoxanthin production. Low light intensity (below 120 μmol photons m^−2^ s^−1^) promotes fucoxanthin production; however, below a certain threshold, it can have a negative impact on microalgae growth [25,26,27]. Light quality, particularly the ratio between blue and red light, was shown to affect pigment profile production in *P. tricornutum* [28,29]. Variations in the blue- and red-light ratio differently affect both fucoxanthin content and microalgae growth; thus, a two-phase culture strategy to maximise growth before inducing the pigment production through a light ratio shift (coupled with tryptone supplementation) has been previously proposed [27].

Regarding the optimisation of media composition for fucoxanthin production, most studies have focused on variations in the nitrogen source and its concentration [25,26,30,31]. Nitrogen is a key nutrient for microalgae growth and is involved in the regulation of the synthesis of cellular metabolites such as fucoxanthin and lipids [2]; thus, the modulation of this macronutrient can lead to enhanced growth and pigment content. Several nitrogen sources have been tested, including nitrate, ammonium chloride, urea and ammonium sulphate [30,32,33]. Nitrate is considered the most efficient nitrogen source since it leads to a high growth rate and increased fucoxanthin production [2] and is stable during autoclaving, facilitating media preparation in the laboratory [34]. Nitrogen media supplementation was found to further increase fucoxanthin productivity [2]. Yeast extract and tryptone supplementation increased fucoxanthin production up to 2-fold in *P. tricornutum* [31]. Similarly, increasing concentrations of sodium nitrate in the media led to enhanced fucoxanthin productivity [25,26]. An approximate 3-fold increase in fucoxanthin concentration was reported when f/2 medium was supplemented with 10-fold sodium nitrate, but this study was carried out under temperatures of 25 ± 4 °C, higher than the temperature typically used for *P. tricornutum* growth [25]. The identification of optimal growth conditions is crucial to guarantee the sustainability of the future large-scale production of fucoxanthin. Therefore, in this work, we investigated optimal sodium nitrate supplementation on *P. tricornutum* growth and fucoxanthin production.

## 2. Materials and Methods

### 2.1. Microalgae Strain and Growth Conditions

*Phaeodactylum tricornutum* strain CCAP 1055/1, from the Culture Collection of Algae and Protozoa of the Scottish Association for Marine Science, UK was grown in f/2 media supplemented with silica (f/2 + Si; basal medium) according to [35]. The non-axenic diatom was grown as 50 mL batch cultures in 250 mL Erlenmeyer flasks at 19 ± 1 °C, in a rotary shaker at 110 rpm (Agitorb 200IC, Aralab, Rio de Mouro, Portugal) under a photosynthetically active photon flux density (PPFD) of 30 µmol photons m^−2^ s^−1^ [27,36] with a 16 h light/8 h dark cycle [32,37]. Subcultures were prepared by adding a 1:3 ratio of inoculum and fresh medium, respectively, under aseptic conditions, in a biosafety laminar flow hood (Faster Bio48, Milan, Italy). In all assays, the initial inoculum originated from cultures grown in medium with the basal concentration of sodium nitrate (0.882 mM).

### 2.2. Analysis of P. tricornutum Growth Curve below Basal NaNO_3_ Concentration

A preliminary study of the growth curves of *P. tricornutum* was carried out for 7 days, in 50 mL of f/2 + Si medium, with a gradual increase in the concentration of NaNO_3_ between 0 and 10-fold basal concentration (0.882 mM NaNO_3_). Within this interval, each concentration is approximately the double of the previous one, as follows: 0, 0.183, 0.220, 0.441, 0.882, 1.764, 3.528, 7.056 and 8.82 mM NaNO_3_ [32,38].

To firstly assess growth, cell counting was performed using a Neubauer chamber (Hirschmann, Neckartenzlingen, Germany), under an upright microscope (Leica DM6 B, Wetzlar, Germany), at 40× magnification. Three biological replicates (corresponding to three Erlenmeyer flasks) were used for each NaNO_3_ concentration.

### 2.3. Analysis of P. tricornutum Growth Curve above Basal NaNO_3_ Concentration

Growth curves for 1×, 10×, 20× and 40× basal concentration of sodium nitrate [32,39] were drawn based on optical density measurements, for convenience. A volume of 600 µL of each 50 mL culture was collected. Samples were centrifuged at 10,000× *g* for 3 min and the supernatant was discarded. The pellet was resuspended in 600 µL of the respective growth medium, and the OD readings at 750 nm were obtained using a 96-well microplate reader (EPOCH2, BioTek, Winooski, VT, USA). Three independent readings of 200 µL per well each were performed.

### 2.4. Extraction and Quantification of Fucoxanthin

*P. tricornutum* cells were centrifuged at 3000× *g* (Allegra X-12R Centrifuge, Beckman Coulter, Brea, CA, USA), and the pellet was rinsed with distilled water and recollected by centrifugation. Cells were frozen in liquid nitrogen and thawed in ice for five minutes; this process was repeated three times. To proceed with pigment extraction, cells were suspended in ethanol (1:30; *m*/*v*), and placed at 45 °C for 2 h, vortexing every 30 min [40]. Finally, extracts were centrifuged at 3000× *g*, and the pigment solution was filtered through a 0.22 µm nylon syringe filter (VWR, Radnor, PA, USA). Samples were protected from light exposure whenever possible. The collected pigment solutions were analysed by high performance liquid chromatography (HPLC—Waters Alliance 2695 System) with a photo diode array 2996 detector with a range of 190 to 800 nm and a C18 reverse phase column (5 µm particle size, 3.9 × 150 nm, Delta-PAK, Lisboa, Portugal). The mobile phase was a linear gradient, with a flow rate of 0.5 mL min^−1^ of acetonitrile:water from 80:20 to 100:0 over 8 min, maintained for 3 min and then reduced back to 80:20 over 3 min. The chromatogram was recorded at 445 nm. Fucoxanthin was identified by retention time, absorption spectra and co-chromatography with a commercially available standard (16337-1MG, Sigma-Aldrich, St. Louis, MO, USA) [40].

The relative quantification of fucoxanthin production can also be obtained by a spectrophotometric method developed by Wang and co-workers [40]. In order to apply the formula described in [40], we firstly established a correlation between UV/Vis spectrophotometer cuvette absorbance (Nanodrop ND-2000C, ThermoFisher, Waltham, MA, USA) and microplate readings, using *P. tricornutum* grown in basal medium. To carry out the measurements, 3.6 mL of culture was collected, centrifuged at 3000× *g* for 5 min, and the pellet was resuspended in basal medium. In parallel, the process was repeated but the pellet was resuspended in equal volume of ethanol. Both suspensions were transferred to cuvettes (1 mL) and 96-well plates (200 µL), and three independent readings were carried out at 750 nm, for *P. tricornutum* resuspended in medium, and at 445 nm and 663 nm for *P. tricornutum* suspended in ethanol. The path length was corrected to 1 cm using the software provided with the microplate reader. The regression lines for A_445_, A_663_ and A_750_ were inferred by plotting the spectrophotometer absorbance readings in the *x*-axis and the microplate readings in the *y*-axis [40]. Cells from each assay were collected and resuspended in basal medium or ethanol, as described above, and three independent readings for each sample were performed in a 96-well plate. The wavelength values obtained from the microplate readings were replaced in the regression equations (y values), and the correlated values (x values) were calculated. With the data collected, the concentration of fucoxanthin was calculated by applying Equation (1).

[Fucoxanthin] mg L^−1^ = 6.39 × A_445_ − 5.18 × A_663_ + 0.312 × A_750_ − 5.27, (1)

where [Fucoxanthin] is the concentration of fucoxanthin in mg L^−1^, A_445_ is the absorbance at 445 nm, A_663_ is the absorbance at 663 nm, and A_750_ is the absorbance at 750 nm. The absorbance values at 445 nm and 663 nm needed to be between 0.2 and 1, while for a 750 nm reading, they needed to be between 0.1 and 0.8 [40]. If the values did not match the indicated intervals, appropriate dilutions were performed.

The specific growth rate was calculated using Equation (2).

μ = ln(x2/x1)/(t2 − t1),(2)

where μ is specific growth rate (d^−1^) and x1 and x2 are the OD750 at time t1 and t2, respectively, according to [41].

Daily volumetric fucoxanthin productivity in *P. tricornutum* (mg L^−1^ d^−1^) was calculated according to [42,43].

### 2.5. Cell Viability Analysis

Cells at day 7 of growth were stained with Neutral Red 0.2% (*m*/*v*) (adapted from [44]) in ddH_2_O, in a 1:10 proportion of staining solution to cell suspension, and incubated for 20 min at room temperature, in the dark. Cell observations were carried out under an optical microscope Leica DM6 B, Wetzlar, Germany, and photographs were taken using a colour DMC4500 camera with Leica LAS X software.

### 2.6. Statistical Analyses

Statistical analyses were performed using SPSS Statistics 28 (IBM Corporation, Armonk, NY, USA). All tests were performed considering the significance level at 5% (*p*-value < 0.05). The Shapiro–Wilk test and Levene’s F-test were used to test normality and variance of homogeneity, respectively. As the data did not meet the assumptions, nonparametric Kruskal–Wallis and Games-Howell tests were used.

## 3. Results

### 3.1. Cell Growth from 0 to 10× Basal Sodium Nitrate Concentration

**Figure 1:**
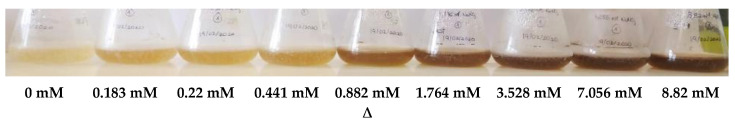
Culture flasks with 50 mL of growth medium supplemented with various concentrations of sodium nitrate, at the end of the 7th day of growth. Concentrations are in ascending order, from left to right, from 0 mM to 8.82 mM. The arrow indicates the basal concentration (0.882 mM), commonly used for the growth of this species.

*P. tricornutum* cells were grown in 250 mL flasks to assess the effect of distinct concentrations of sodium nitrate, below and above the basal concentration of 0.882 mM that is commonly used in the f/2 medium. In a first approach, culture growth was followed for 7 days, during which cells were counted, to follow the increase in biomass more closely. Visual results can be clearly observed in Figure 1, in which the dark brown colour intensifies as the sodium nitrate concentration in the media increases.

**Figure 2:**
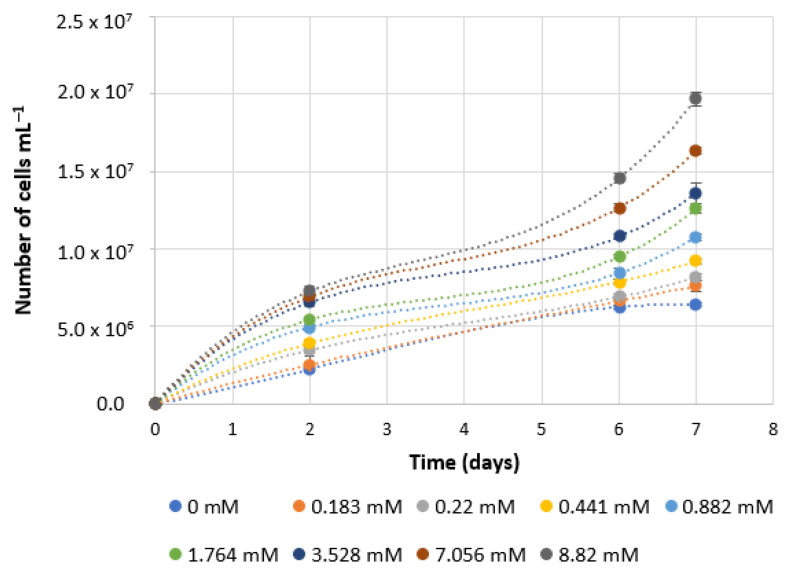
Cell number in cultures of *Phaeodactylum tricornutum* grown in 250 mL flasks with 50 mL of culture medium, supplemented with various concentrations of sodium nitrate. Cell counts were performed on days 0, 2, 6 and 7. The number of cells is represented as the mean of cells per millilitre ± standard deviation (*n* = 3), normalised to zero at day 0. Dotted lines represent trend adjustments, in the form of polynomial equations.

Analysis of the growth of *P. tricornutum* cultures under the above-mentioned conditions is shown in Figure 2. Increasing concentrations of NaNO_3_ led to higher cell numbers during the time period of 7 days under study. Thus, concentrations of sodium nitrate up to 10× higher than the ones commonly used may result in more biomass per litre of medium and may prove advantageous. These results point to a potential gain when increasing the medium with the NaNO_3_ concentration, compared to the basal concentration.

### 3.2. Cell Growth from 10× to 40× Basal Sodium Nitrate Concentration

**Figure 3:**
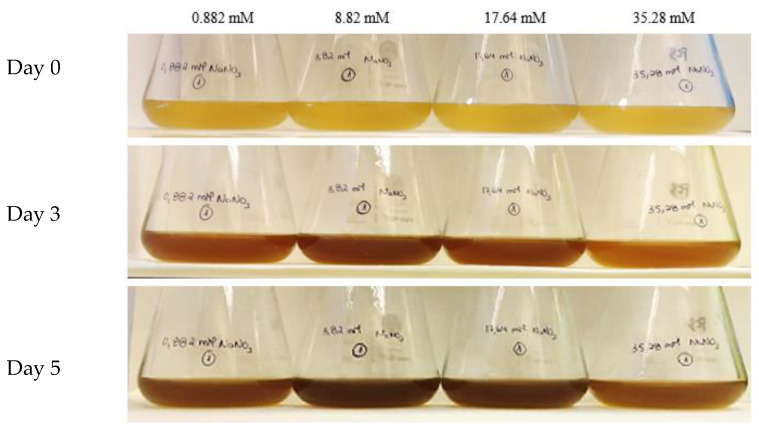
Flasks with 50 mL of culture medium (basal 0.882 mM), 10× (8.82 mM), 20× (17.64 mM) and 40× (35.28 mM) of NaNO_3_. Photos taken on day 0, and on days 3 and 5 after inoculation.

Having previously found that a 10-fold increase in sodium nitrate concentration led to higher cellular yields, we set out to investigate whether cell growth (and possibly fucoxanthin accumulation) would further increase at higher concentrations of sodium nitrate, and whether any adverse effects would occur. To this end, we tested 10×, 20× and 40× concentrations of NaNO_3_ comparatively with the basal f/2 medium. Visual aspects of the cultures can be seen in Figure 3.

**Figure 4:**
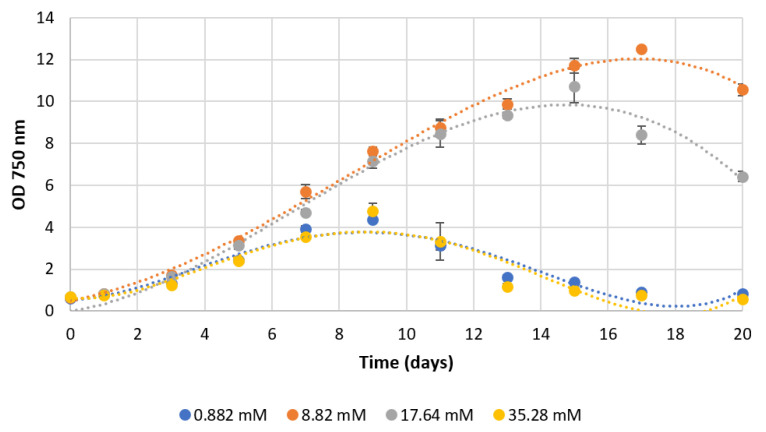
Growth curves of *Phaeodactylum tricornutum* (OD_750 nm_), as a function of the amount of nitrate (NaNO_3_) present in the culture medium, along 20 days of growth. Data are mean values ± standard deviation (*n* = 3). Dotted lines represent trend adjustments, in the form of polynomial equations.

Figure 3 clearly shows that the culture exhibiting the darkest colour corresponds to sodium nitrate concentration of 8.82 mM (10× basal medium), which seems to indicate that higher concentrations (20× or 40×) would not be advantageous. To confirm this, cultures were followed over an extended period of 20 days and growth was monitored by measuring the OD at 750 nm (Figure 4). In this case, instead of cell number, for convenience, we used the optical density value at 750 nm as an estimate of cell biomass concentration.

The results show that in basal concentrations of NaNO_3_, cell density reaches a peak at day 9 after initial inoculation. From day 9 onwards, we witnessed a decrease in OD, revealing a pattern of cell death. When NaNO_3_ molarities in the range of 35.28 mM are used, the pattern is very similar and there are no gains or adverse effects on growth compared to the standard conditions. Interestingly, at intermediate values of 8.82 mM (10×) and 17.64 mM (20×), gains in cell density are visible, reaching highest values at day 17 for the 10× supplemented cultures. The biomass peak in the 17.64 mM (20×) condition is reached earlier, by day 15, but shows lower values. Apparently, 10× to 20× magnification of nitrate concentration leads to an extension of the growth period of the cultures but allows them to reach higher cell density values. The statistical tests indicate that from day 7 of culture, there are statistically significant differences (*p* < 0.05) between the data obtained in the standard culture (0.882 mM) and the supplementation with 10× and 20× sodium nitrate. By day 5, the supplementation with 10× nitrate already indicates significant differences relative to the standard condition (*p* < 0.05). As the use of specific growth rate calculation is common in microalgae, including in *P. tricornutum* [45], we also calculated the respective values (Table 1), between the third and the eleventh day after inoculation.

### 3.3. Fucoxanthin Production from 0× to 10× Standard Sodium Nitrate Concentration

**Figure 5:**
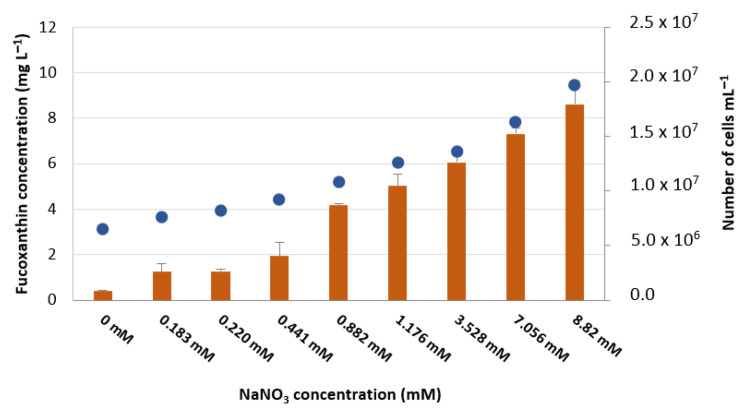
Fucoxanthin total production in *Phaeodactylum tricornutum* (mg L^−1^), as a function of the amount of nitrate (NaNO_3_) present in the culture medium, after 7 days of growth (orange bars) and average number of cells in the same conditions (blue dots). Values were obtained by HPLC. Data are mean values ± standard deviation (*n* = 2).

As our goal was to adjust the sodium nitrate content of the basal medium F/2, to maximise fucoxanthin production in *P. tricornutum*, we analysed and quantified fucoxanthin content in distinct conditions under study. The results obtained in the initial tests, for 7 days, are represented in Figure 5. Clearly, fucoxanthin concentration increases directly with the nitrate concentration used, in the range of values under analysis. This effect is only partially due to the increase in cell density in culture, but it is also due to an increase in productivity per cell.

### 3.4. Fucoxanthin Production from 10× to 40× Basal Sodium Nitrate Concentration

**Figure 6:**
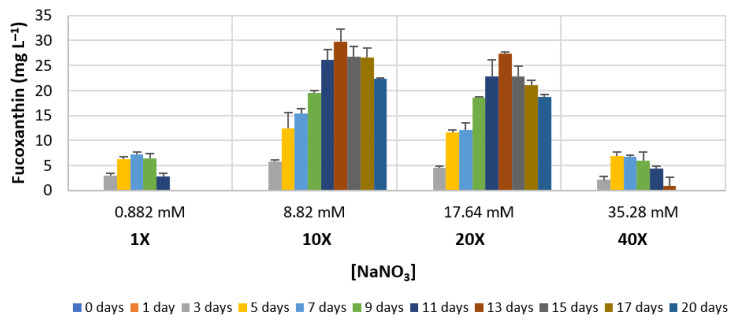
Fucoxanthin production in *Phaeodactylum tricornutum* (mg L^−1^), as a function of the amount of nitrate (NaNO_3_) present in the culture medium, along 20 days of growth. Data are mean values ± standard deviation (*n* = 3).

Fucoxanthin volumetric concentration was determined for each culture condition, using OD readings and applying an adapted version of the formula by [40]. Figure 6 shows the amount of fucoxanthin in mg per litre, when supplementing the basal medium with 10×, 20× and 40× sodium nitrate. This quantification was performed during a 20-day period. The results indicate a clear increase in the volumetric production of fucoxanthin when the medium is supplemented with NaNO_3_ increased by 10× and 20×, with maximum values at day 13. In the basal condition, the maximum of 7.16 mg L^−1^ is reached after 7 days, a much lower value than that achieved in the 10× and 20× supplementation, with 29.69 and 27.38 mg L^−1^, respectively. It is also worth noting that when the nitrate concentration is increased by 40×, a maximum volume productivity value of 6.79 mg L^−1^ (day 5) is observed; these values are comparable to the basal medium to which there are no significant differences. The statistical analysis through the Kruskal–Wallis test and pair wise comparison showed significant differences (*p* < 0.05) from day 5 onwards, when 10× and 20× conditions were compared with the basal medium (0.882 mM NaNO_3_).

**Figure 7:**
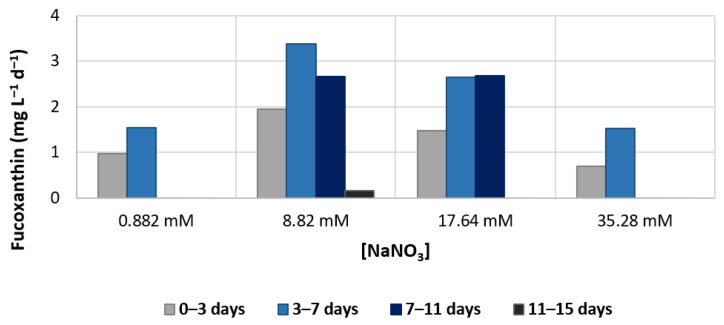
Daily volumetric fucoxanthin production in *Phaeodactylum tricornutum* (mg L^−1^ d^−1^), as a function of the amount of nitrate (NaNO_3_) present in the culture medium, along the first 15 days of growth. Data are mean values ± standard deviation (*n* = 3).

The daily productivity was also calculated, and the results are shown in Figure 7. Here, it is visible that the maximum increase occurs with NaNO_3_ supplemented with 10× basal concentration between day 3 and day 7. Between day 7 and day 11, there are no differences in the rate of increase between medium with 10× and 20× NaNO_3_.

### 3.5. Assessment of Cell Viability under Distinct Nitrate Concentrations

**Figure 8:**
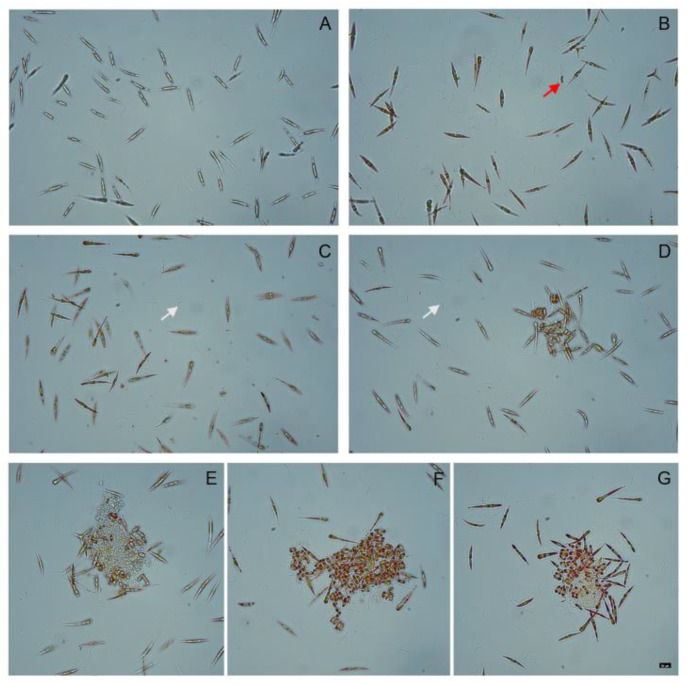
Images at day 7 of growth stained with neutral red. Cultures supplemented with 0.882 mM (**A**), 8.82 mM (**B**), 17.64 mM (**C**) and 35.28 mM (**D**) of NaNO_3_. White arrow indicates bacteria and red arrow indicates a dead cell. (**E**–**G**) Images of cell aggregates of *P. tricornutum* supplemented with 35.28 mM of NaNO_3_ (40× basal nitrate). Bar 10 µM, all photos were taken with the same magnification.

Visual observation of the cell viability of the cultures under distinct nitrate concentrations was carried out using neutral red. Figure 8 shows microscopic images of the cells at the seventh day of growth, under four nitrate concentrations. Higher concentrations of nitrate seemed to cause cell aggregates with a prevalence of round cells in the centre of the cluster. Cells growing under 40× basal nitrate tend to aggregate around a matrix that is clearly visible in panels E, F and G. This matrix is likely to correspond to what is described as extracellular polymeric substances (EPSs) that are formed under stressful conditions and are further discussed below.

## 4. Discussion

In this report, the growth kinetics of *P. tricornutum* cultures on f/2 medium was studied, considering variations in nitrogen content from 0 to 40× basal concentration. The standard medium currently used for this diatom species contains nitrogen in the form of sodium nitrate, at the concentration of 0.882 mM. Our aim was to study the effects of varying this compound concentration on cell growth and fucoxanthin production, looking to achieve the optimisation of NaNO_3_ concentration in the f/2 medium for maximum production.

Fucoxanthin is a pigment of marine origin, present in brown algae and diatoms, whose beneficial properties are widely known [23,46]. Although most of the studies focus on its beneficial effects on human health, these properties also allow us to envisage the use of these compounds in human nutrition, particularly their inclusion in so-called functional foods [47]. These foods are increasingly present in consumers’ minds, thus pressuring the food industry to offer innovative solutions which are safe and have proven benefits [48]. Studies indicate that the incorporation of fucoxanthin in foods should preferably be carried out via encapsulation or incorporation, in solutions with a pH range of 5 to 7, with storage at 4 °C or room temperature, in the dark [23]. These conditions guarantee the stability and functionality of the compound. Thus, considering the studies available to date, fucoxanthin has become a very promising component for inclusion in the formulation of functional foods. The potential applications of fucoxanthin are very clear; however, there are limitations related to the commercial availability of this compound, which is almost non-existent in its purified form.

Direct extraction from marine organisms should be optimised, thus allowing one to increase yields and reduce production costs while providing a natural and safe product. However, one of the factors that still deserves attention is the primary production of fucoxanthin, based on organisms such as the model diatom *P. tricornutum*, using approaches in which the growth conditions are manipulated and the production yield is maximised. These strategies of adjusting cultivation conditions offer the potential for gains without adding significant costs at the production level.

Previous studies have looked at different culture conditions of *P. tricornutum*, modifying light intensity and wavelength, medium composition or CO_2_ addition, among other factors [25,49]. Fucoxanthin, like other carotenoid pigments, undergoes changes in its levels, mainly because of changes in luminosity (light intensity and quality) and nitrogen availability. Interestingly, nitrogen deficiencies in some species can result in higher levels of some pigments, presumably by generating a stress situation. The classic example is the production of β-carotene in *Dunaliella salina*, where a first phase occurs, with optimum conditions for the obtention of biomass, followed by exposure to stress conditions, by high light intensity and nitrogen reduction, which maximise the production of the pigment of interest. Previous studies from McClure and colleagues [25] showed that fucoxanthin production in *P. tricornutum* in f/2 medium, with light intensity above 100 μmol photons m^−2^ s^−1^, and the addition of nitrate in the culture medium, had a higher biomass yield. The growth temperature (25 ± 4 °C), light intensity, the strain used (CS-29 from ANACC), the bioreactor type and the injection of CO_2_ were quite different conditions from those used in the present study, which makes it difficult to directly compare results. However, generally, the results indicated that a light intensity of 150 μmol photons m^−2^ s^−1^, the supplementation of f/2 medium with nitrate (10×), did not result in an increase in the specific growth rates in the first 7 days, but it resulted in significant increases in the amount of fucoxanthin obtained, particularly at day 9 of growth, with the maximum volumetric concentration of fucoxanthin of 20.5 mg L^−1^. This result was obtained with the injection of 1% (*v*/*v*) of CO_2_. Thus, as already reported by other authors, cell productivity may not necessarily be aligned, in terms of optimal cultivation conditions, with maximum fucoxanthin yield, and further studies must be carried out.

Nitrogen metabolism in diatoms is unique and has not yet been fully unravelled [50]. There are, however, already studies concerning *P. tricornutum* that try to unveil some aspects, based on transcriptomics, proteomics and metabolomics tools. Diatoms can assimilate nitrogen from various sources, notably nitrate, but also ammonia and urea. The assimilation of nitrate and ammonia is usually carried out via specific transporters that are up regulated in situations of ammonia excess. When assimilated by the cells, nitrate is converted into nitrite and directed to the chloroplast, where it is converted into ammonia [51]. The ammonia then enters the GSII-GOGAT cycle, producing glutamine and glutamate, which are used to meet the cell’s nitrogen needs [50,52]. The growth inhibition we observed in culture medium with high nitrate concentration (40×) might be related to a process of assimilation and regulation of nitrate levels similar to the one reported in *Chlorella vulgaris* [39]. Increasing the nitrate concentration in the medium induces an increase in nitrate reductase activity at the cellular level, resulting in the accumulation of ammonia, which in turn may explain the reduction in growth. The accumulation of ammonia may trigger upstream regulatory processes and inhibit nitrate assimilation, as nitrate assimilation is quickly blocked by ammonium addition, leading in turn to a slowdown in growth rates [51]. In parallel, the accumulation of nitrite in the cells can also have toxic effects. In *Spirulina platensis*, growth was severely inhibited by increasing ammonia concentration under autotrophic conditions [53], and negative effects on photosynthetic pigment content were recorded.

Our results show that the maximum volumetric concentration of fucoxanthin occurred on the seventh day, in f/2 basal medium, with a value of 7.16 mg L^−1^; however, in medium where the amount of nitrate was increased by 10-fold, the maximum value was recorded later, by the thirteenth day, with a value of 29.69 mg L^−1^. On the ninth day of growth, we recorded values very similar to those of McClure et al. [25] with 19.45 mg L^−1^. Despite the differences between the methodologies used and the fact that in our study cultures were not supplemented with CO_2_, we found higher maximum production of fucoxanthin (50% increase). Other authors also reported that the maximum yield of fucoxanthin in *P. tricornutum* occurred on the fifth day of growth (2.42 mg g^−1^ of dry biomass), with a maximum specific growth rate (1.91 day^−1^) on day 4 [54]. These values, such as those found in our studies, occur during the exponential growth phase. In our study, the maximum specific growth rate, for the standard conditions, occurred between days 3 and 5. Apparently, our growth conditions allow the cells to multiply faster and reach a higher volumetric concentration of fucoxanthin.

*P. tricornutum* under nitrogen stress performs a proteomic change, where it reduces the use of nitrogen and redirects metabolic pathways towards reducing the photosynthetic apparatus, including the pigments that are part of it, and potentiating the synthesis of triacylglycerols [55,56]. The genes involved in fucoxanthin biosynthesis show a marked reduction in their expression levels, being, together with the genes involved in chlorophyll biosynthesis, the most downregulated. The study by Alipanah et al. [57] reports that by day 3 of growth, differentiation emerges between cultures growing on f/2 medium, which are either at normal nitrogen levels or in deficiency. In our case, this difference is only significant from day 5 onwards (Figure 4), but the different units make direct comparison difficult. The authors [57] found that the amount of fucoxanthin (fg cell^−1^), at 48 h of growth, doubled its value in cells grown in the presence of nitrate, compared to cells without nitrogen. Other authors reported negative effects of nitrate reduction in the medium, namely the studies by Megía-Hervás and collaborators [58], who investigated the effect of the presence of sodium nitrate, at concentrations of 0 mM, 5.9 mM and 11.8 mM, on the biomass yield of *P. tricornutum*. These authors observed that in medium with nitrate concentration of 11.8 mM, the stationary phase was reached at day 10 of growth, and nitrate was depleted at day 13. A reduction of nitrate to 5.9 mM brings forward the stationary phase due to nitrogen limitation. However, the authors did not test nitrate concentrations above 11.8 mM, nor did they investigate fucoxanthin levels.

In our experiments, the amount of fucoxanthin determined at 7 days of growth was 4.17 mg L^−1^ in basal medium (with 0.882 mM of NaNO_3_), which means a 10-fold increase compared with nitrate deficiency conditions (with 0.4 mg L^−1^ in medium without NaNO_3_). Moreover, when the amount of nitrate increases to 8.82 mM (Figure 5), the amount of fucoxanthin increases to a value of 8.62 mg L^−1^, doubling the values obtained in basal medium. This clearly states that the increase in nitrogen is an important factor to allow the maximum fucoxanthin production yield. The addition of other forms of nitrogen, such as KNO_3_, even in different systems, also allow one to obtain values of volumetric productivity of fucoxanthin, which are maximum at day 6 of growth, with a value of 4.73 mg L^−1^ d^−1^ [59]. In the study, the authors used a 55 L flat-plate glass photobioreactor system and 14.5 mmol L^−1^ of KNO_3_ and demonstrated that the reduction of nitrogen in the media always led to a reduced volumetric productivity of fucoxanthin, regardless of the days. The values we obtained were maximum at day 7, with 3.37 mg L^−1^ d^−1^, which are lower than the ones found by these authors, but again, the growth systems and strain are distinct.

Gómez-Loredo and coworkers [60] noted that the use of agitation instead of aeration may be a difficulty in obtaining high growth rates in *P. tricornutum*. The authors point out that cultures under orbital agitation take about 2 days to make the start of exponential growth, which slows down after 8 days. The authors also state that the highest cell densities were not reached in the same conditions where the highest fucoxanthin concentration was observed, and so care must be taken when analysing the results. In fact, the authors stated that *P. tricornutum* cultures showed the maximum concentration of fucoxanthin (about 0.2 mg g^−1^ FW) on the eighth day in aerated conditions and light intensity of 13.5 μmol photons m^−2^ s^−1^. The authors further indicated that cultures with agitation and a luminous intensity of 25.9 μmol photons m^−2^ s^−1^ (similar to the value of 30 μmol photons m^−2^ s^−1^ used in the present study) always resulted in lower values of both biomass and fucoxanthin productivity compared to lower light intensities. The choice of the growth temperature of 19 ± 1 °C took into account results previously obtained by other authors [61], who obtained specific growth rate values of 0.87 ± 0.034 (d^−1^) when growth was performed at 20 ± 2 °C, even though this species grows in a wide range of temperatures [62].

Interestingly, we also observed cellular morphological changes as the content in nitrate increased in the culture medium. At 40× basal concentration, cells tended to aggregate around a matrix of presumably extracellular polymeric substances or EPS (Figure 8E–G). The centre of these clusters contained round-shaped cells, as opposed to the fusiform morphotype that was prevalent in *P. tricornutum* cell cultures with lower nitrate concentrations. Microscopy observation of these cultures seemed to indicate an increase in the number of bacteria as nitrate concentrations increased, although this was not quantified. To gain further insight, we carried out a Mass Spectrometry analysis of the proteome of the spent medium and found a prevalence of proteins from *Roseobacter*, which is in line with other reports available in the literature [63]. These authors report that a marine *Roseobacter* strain influences the aggregation of *P. tricornutum* by inducing a morphotypic transition from fusiform to oval shape. These bacteria, which live closely associated with microalgae, influence their growth, aggregation and secretion of exopolymers [63]. Depending on growth conditions, *P. tricornutum* may change its appearance between triradiate, fusiform and oval cell morphologies, of which the latter is usually related with a stress or defence response. Although the cellular behaviour of *P. tricornutum* is not the main focus of this report, it is worth noticing that it can be correlated with the metabolic changes discussed above. In fact, previous studies aiming to optimise the medium composition in order to increase EPS production highlight the role of sodium nitrate. It has been reported that increasing nitrogen concentration results in elevated EPS production (reviewed in [64,65]), although this is not consensual, and some authors have observed opposing effects [66]. Further studies need to be carried out in order to fully understand the intricate relationship between microalgae communities (including associated bacteria), nitrogen usage and response to challenging environmental conditions.

Overall, this work provides evidence that there is a gain in increasing the established standard nitrate concentration present in culture media when the goal is to produce fucoxanthin in a profitable way. The precise amount of nitrate to be added needs to be determined for each cultivation condition, as it is evident that distinct parameters lead to different results, namely CO_2_, temperature and light conditions. If the goal is to produce fucoxanthin on a large scale at the industrial level, then the balance between the cost of the nitrate source and the resulting pigment yield needs to be properly evaluated, keeping in mind that these cultures are usually non-axenic, and all organisms of the community contribute to the final outcome.

## Figures and Tables

**Table 1:** *Phaeodactylum tricornutum* specific growth rates (*µ*), between days 3 and 11, in different time intervals (d, days), as a function of the amount of nitrate (NaNO_3_) present in the culture medium.

	*µ* (d^−1^)				
[NaNO_3_]	1–3 d	3–5 d	5–7 d	7–9 d	9–11 d
0.882 mM	0.265	0.304	0.237	0.057	−0.166
8.82 mM	0.368	0.336	0.268	0.144	0.068
17.64 mM	0.356	0.317	0.203	0.209	0.084
35.28 mM	0.242	0.333	0.200	0.146	−0.181

## Data Availability

The data presented in this study are available on request from the corresponding author (R.A.), upon reasonable request.
